# Impact of a Virtual Culinary Medicine Curriculum on Biometric Outcomes, Dietary Habits, and Related Psychosocial Factors among Patients with Diabetes Participating in a Food Prescription Program

**DOI:** 10.3390/nu13124492

**Published:** 2021-12-15

**Authors:** Shreela V. Sharma, John W. McWhorter, Joanne Chow, Melisa P. Danho, Shannon R. Weston, Fatima Chavez, Laura S. Moore, Maha Almohamad, Jennifer Gonzalez, Esther Liew, Denise M. LaRue, Esperanza Galvan, Deanna M. Hoelscher, Karen C. Tseng

**Affiliations:** 1 Department of Epidemiology, Human Genetics & Environmental Sciences, Michael & Susan Dell Center for Healthy Living, University of Texas School of Public Health, 1200 Pressler, Houston, TX 77030, USA; Joanne.W.Chow@uth.tmc.edu (J.C.); Melisa.P.Danho@uth.tmc.edu (M.P.D.); Fatima.Chavez@uth.tmc.edu (F.C.); Maha.Almohamad@uth.tmc.edu (M.A.); 2 Department of Health Promotion Behavioral Sciences, Michael & Susan Dell Center for Healthy Living, University of Texas School of Public Health, 1200 Pressler, Houston, TX 77030, USA; John.Wesley.Mcwhorter@uth.tmc.edu (J.W.M.); Shannon.R.Weston@uth.tmc.edu (S.R.W.); Laura.S.Moore@uth.tmc.edu (L.S.M.); 3 Population Health, Harris Health System, 4800 Fournace Place, Bellaire, TX 77401, USA; Jennifer.Gonzalez@harrishealth.org (J.G.); Denise.LaRue@harrishealth.org (D.M.L.); Esperanza.Galvan@harrishealth.org (E.G.); Karen.Tseng@harrishealth.org (K.C.T.); 4 Food For Change Health Partnerships, Houston Food Bank, 535 Portwall Street, Houston, TX 77029, USA; Eliew@houstonfoodbank.org; 5 Department of Health Promotion Behavioral Sciences, Michael & Susan Dell Center for Healthy Living, Austin Regional Campus, University of Texas School of Public Health, 1616 Guadalupe, Austin, TX 78701, USA; Deanna.M.Hoelscher@uth.tmc.edu

**Keywords:** self-efficacy, food prescription programs, diabetes, healthy eating, culinary medicine

## Abstract

Culinary medicine is an evidence-based approach that blends the art of cooking with the science of medicine to inculcate a healthy dietary pattern. Food prescription programs are gaining popularity in the Unites States, as a means to improve access to healthy foods among patient populations. The purpose of this paper is to describe the implementation and preliminary impact of A Prescription for Healthy Living (APHL) culinary medicine curriculum on biometric and diet-related behavioral and psychosocial outcomes among patients with diabetes participating in a clinic-led food prescription (food Rx) program. We used a quasi-experimental design to assess APHL program impact on patient biometric outcome data obtained from electronic health records, including glycosylated hemoglobin (HbA1c), body mass index (BMI), and blood pressure (*n* = 33 patients in the APHL group, *n* = 75 patients in the food Rx-only group). Pre-post surveys were administered among those in the APHL group to monitor program impact on psychosocial and behavioral outcomes. Results of the outcome analysis showed significant pre-to-post reduction in HbA1c levels among participants within the APHL group (estimated mean difference = −0.96% (−1.82, −0.10), *p* = 0.028). Between-group changes showed a greater decrease in HbA1c among those participating in APHL as compared to food Rx-only, albeit these differences were not statistically significant. Participation in APHL demonstrated significant increases in the consumption of fruits and vegetables, fewer participants reported that cooking healthy food is difficult, increased frequency of cooking from scratch, and increased self-efficacy in meal planning and cooking (*p* < 0.01). In conclusion, the results of our pilot study suggest the potential positive impact of a virtually-implemented culinary medicine approach in improving health outcomes among low-income patients with type 2 diabetes, albeit studies with a larger sample size and a rigorous study design are needed.

## 1. Introduction

In 2018, 13% of all adults in the United States (US) suffered from type 2 diabetes, with rates increasing by increasing age and being disproportionately higher among Hispanic and African Americans [1]. Consequently, adults with diabetes carry a 50% higher risk of mortality and spend more than $9500 yearly on medical costs than their counterparts, in addition to increased risk of complications and co-morbidities [1,2]. Alarmingly, the prevalence of diabetes is forecasted to increase by more than 50% over the next decade [2]. Substantial evidence demonstrates the relationship between a healthy dietary pattern rich in fruits, vegetables, and whole grains and a lower risk of chronic diseases, including type 2 diabetes [3]. Despite well-supported studies, programs, and public health campaigns presenting the health benefits of consuming a healthy dietary pattern, most Americans, especially those from low-income populations, fall far short of the recommendations [4]. Further, food insecurity and diabetes often co-exist within the same individual because of disordered eating, consuming when and what is available regardless of whether the foods are healthy [5]. The compounding effects of the social determinants of health, such as poverty, food insecurity, lack of access to healthcare, and consumption of energy dense and nutrient-deficient foods continue to negatively affect chronic disease health outcomes [6].

Harris Health system is a fully-integrated safety net healthcare system that provides care for all people residing in Harris County, Texas, regardless of their health insurance status. Harris Health clinics and hospitals serve the most impoverished geographic areas in Harris County, with residents suffering significantly higher rates of food insecurity, overweight and obesity, and type 2 diabetes [7]. More than 55% of the patients served by Harris Health live below the poverty line; and in 2018, the prevalence of overweight and obesity among Harris Health adult patients aged 18 to 44 was 73.9%, which is higher than the corresponding rates for Harris County (68.9%) [8] (Harris Health, personal communication). In 2018, to address health disparities, Harris Health began screening all patients for food insecurity using the two-item Hunger Vital Sign [9], and launched an initiative in collaboration with the Houston Food Bank to install food pantries called “food farmacies” and implement a food prescription framework onsite in select Harris Health ambulatory clinics and hospitals. As part of this strategy, patients screened to be positive for food insecurity and diagnosed with a diet-related chronic disease (e.g., diabetes) are given a 9-month prescription that they can redeem bi-weekly in the “food farmacy” clinic for healthy foods, such as fruits and vegetables, lean protein, legumes, and whole grains. Subsequently, in 2019, as part of a collaborative project called A Prescription for Healthy Living (APHL) between the University of Texas Health Science Center at Houston (UTHealth) School of Public Health, Harris Health, and Houston Food Bank, registered dietitian nutritionists (RDNs) employed at the Harris Health systems were trained in culinary medicine, and patients with diabetes enrolled in the food prescription program (called “food Rx”) were offered a culinary medicine program. The culinary medicine curriculum, developed and implemented by UTHealth School of Public Health investigators, provides experiential culinary medicine training to RDNs and patients to address the linkages between food insecurity, food systems, dietary consumption, health promotion, and chronic disease prevention and treatment [10]. The results of the needs assessment and description of the RDN training and impact are published elsewhere (McWhorter et al., under review). The purpose of this paper is to describe the implementation and preliminary impact of the culinary medicine curriculum on biometric and diet-related behavioral and psychosocial outcomes among Harris Health patients with diabetes participating in the food Rx program.

## 2. Materials and Methods

### 2.1. Study Design

To conduct outcome analysis for the biometric data, including glycosylated hemoglobin (HbA1c), body mass index (BMI), and blood pressure, we used a quasi-experimental design with the A Prescription for Health Living (APHL) participants as the intervention group (food Rx + culinary medicine), and participants who received the food prescription only (no culinary medicine education) from the same clinic in 2019–2020 as the comparison group. To conduct outcome analysis of the diet-related behavioral and psychosocial factors, we used a one-group pre-post evaluation design among patients participating in the APHL program. This study was approved by the University of Texas Health Science Center Committee for Protection of Human Subjects. Informed consent was obtained from all participants who participated in the APHL program.

### 2.2. Recruitment

For this project, participants were recruited from the Harris Health System’s Strawberry Health Center (“Strawberry Clinic”). Eligibility criteria: (a) current patient of Strawberry Clinic, (b) screened “positive” for food insecurity on the food insecurity screener, (c) hemoglobin A1C (hemoglobin with attached glucose) level of greater than 7%, (d) participating in the food prescription program (food Rx) at the Strawberry Clinic. As part of the 9-month food Rx program, patients received a bi-weekly redemption of 30 pounds of fruits and vegetables and other healthy items, such as lean protein, low-fat dairy, and whole grains (at no cost) at a co-located food pantry in the Strawberry Clinic. Once patients were identified as eligible, they were contacted by Harris Health community health workers via phone and offered the APHL program. A total of 42 patients consented to participate in the APHL program and survey measures. Of these, *n* = 41 patients completed the baseline survey (97% baseline survey completion rate), *n* = 35 patients participated in the APHL program (83% participation rate) of which *n* = 27 attended 4 + sessions, and *n* = 29 patients completed the post-intervention survey (70% post-intervention survey completion rate among those who completed baseline measures).

### 2.3. A Prescription for Healthy Living Culinary Medicine Program Description

An intervention mapping™ [11] process was utilized to systematically map the patient curriculum objective constructs and the desired outcomes for APHL program components. The APHL program consists of two primary components: (1) food provision where participating patients receive a nine-month prescription for thirty pounds of fresh produce and other healthy items, such as whole grains, lean meats, and legumes, and (2) a five-session culinary medicine-based education. Table 1 presents the intervention mapping framework for APHL.

Culinary medicine is an evidence-based approach that blends the art of cooking with the science of medicine to inculcate a healthy dietary pattern [12,13]. This strategy helps participants overcome barriers and improve adherence to healthy dietary patterns through the utilization of experiential culinary nutrition education [14,15]. Culinary medicine adds to current nutrition interventions by incorporating both the practical hands-on preparation and pleasure of food and the scientific knowledge of how nutrition and dietary patterns affect health, especially diabetes prevention [15,16]. The APHL culinary medicine curriculum is grounded in the social cognitive theory [17] constructs, including: (1) outcome expectations that healthy food tastes good and is affordable, simple to prepare, and relevant to all cultures; (2) knowledge and awareness about healthy eating, including the common strategies to overcome common barriers of cost, time, flavor, and skills; (3) self-efficacy for engaging in healthy eating; (4) skills to prepare healthy and flavorful foods; (5) subjective norms that healthy eating is the norm for other representative patients; (6) social support to shop, prepare, and consume healthy foods (vegetables, fruits, whole grains, legumes) and to make each plate representative of the MyPlate; (7) change in cooking, meal planning behaviors that results in improved consumption of healthy foods, and each plate consumed represents MyPlate ratios (Table 2).

### 2.4. Program Pivot Due to COVID-19

The timeline for APHL implementation among patients was scheduled to begin April 2020. However, in March 2020, when the COVID-19 pandemic reached Harris County, it resulted in an immediate shelter-in-place, implementing stringent social distancing and mitigation strategies in the community. As a result, Harris Health rapidly transitioned to deployment of telehealth to provide care virtually to patients, which resulted in the adaptation of our entire APHL curriculum for virtual implementation. This adaptation process conducted between April–June 2020 included the professional development of new videos demonstrating culinary skills, protocol development, and training of our project staff and Harris Health staff for recruitment, retention, and implementation of virtual group education classes. Between June and November 2020, we deployed and successfully implemented five virtual patient cohorts for APHL (described in detail below).

### 2.5. APHL Virtual Sessions Description

The purpose of APHL is to provide patients with diabetes nutrition education, culinary skills, and cooking techniques to increase vegetable consumption (utilizing produce from the food Rx program) and provide a place for participants to find a community to act as a support system through the entire program and beyond. For the virtual sessions, program delivery was a mix of asynchronous and synchronous. For example, the facilitated group discussion for each session topic was conducted synchronously using Webex at a scheduled time and day. The program consisted of bi-weekly five sessions and each session lasted 90 min. Participants had access to virtual cooking videos, resources, recipes, and handouts for each session in English and Spanish asynchronously via project website (see https://sph.uth.edu/research/centers/dell/prescription-for-healthy-living/) (accessed on 20 June 2021). In addition, hard copies of all recipes and handouts were provided at the Food Farmacy for participants when they pick-up their produce. Table 2 outlines the APHL curriculum.

### 2.6. Data Collection Measures

Socio-demographic measures were obtained from the Harris Health EHR (age, gender, race/ethnicity). Food insecurity status was also obtained at baseline from the Harris Health EHR. Harris Health measures food insecurity status system-wide using the two-item Hunger Vital Sign measures [9].

#### 2.6.1. Biometric Outcomes

Biometric data were abstracted by Harris Health staff from the patient electronic medical records. Participants at Strawberry Health Center who received APHL (i.e., food Rx + culinary medicine) were those in the intervention group, and participants who received the food prescription only but did not participate in the culinary medicine education from the same clinic in 2019–2020, as the comparison group. Data abstraction was conducted for glycosylated hemoglobin (HbA1c), systolic and diastolic blood pressure, and body mass index (BMI) computed using height and weight to determine obesity status. For each patient in the intervention group, for baseline measures, data abstraction was conducted if measures were within a 90-day window prior to start, or within 14 days of enrollment into the program. Post-program measures were the values recorded for the biometric variable of interest at the timepoint most proximal to end of program date, within a 90-day post-program time period. Participants whose values were missing in the EHR were classified as lost to follow-up. Comparison group participants were all those patients who participated in the food prescription program only, in a similar time frame as those in the intervention group.

#### 2.6.2. Behavioral and Psychosocial Outcomes

Pre- and post-self-reported surveys were collected to evaluate program impact on diet and psychosocial behaviors.

1. Vegetable consumption was measured using one item, “How many servings of vegetables do you eat or drink each day?”. Response options (0 to 5): none to 4 + servings per day [18];
2. Fruit consumption was measured using one question, “How many servings of FRUIT do you eat or drink each day?”. Response options (0 to 5): none to 4 + servings per day [18];
3. Whole grain consumption was measured using one item, “How many servings of Whole Grains do you eat each day?”. Response options (0 to 5): none to 4 + servings per day [18];
4. Typical frequency of consumption of various foods was measured using the previously validated 7 items, e.g., “How often do you typically eat a green salad?”. Response options (0–4): not at all to more than once a day [19]. Items were assessed individually and as a summative scale;
5. Grocery shopping, meal preparation, and cooking behaviors were measured using 10 items from a previously validated survey, e.g., “How often do you compare prices before you buy food?”. Response options (1–5): never to always [19]. All items were assessed individually;
6. Changes in self-efficacy in cooking and meal planning behaviors were measured using previously validated scale of 5 items, e.g., “Before this program how sure were you that you could use basic cooking techniques (e.g., microwaving, sautéing, roasting)”. Response options (0–4): not at all sure to extremely sure [20]. Items were assessed individually and as a summative scale;
7. Perceived barriers to eating fruits and vegetables were measured using a previously validated scale of 13 items, “E.g., I don’t eat fruits and vegetables as much as I like to because they cost too much”. Response options (0–4): strongly agree to strongly disagree [20]. Items were assessed as a summative scale;
8. Perceptions regarding healthy eating were measured using four items (not validated), “Cooking healthy food is difficult”. Response options (1–5): strongly agree to strongly disagree. Items were assessed as a summative scale;
9. Nutrition knowledge was measured using one item (not validated), “When thinking about preparing a plate of food, how much of your plate should be filled with fruits and vegetables?”. Response options consisted of pictures of MyPlate with one-fourth, half, and three-fourths of the plate being fruits and vegetables;
10. Perceived health was measured using one question, “Overall, how would you rate your health in the past four weeks?”. Response options (1–6): excellent to very poor [21].

### 2.7. Process Evaluation Data

Session dosage, reach, fidelity, and acceptability were conducted using attendance logs and individual session comment cards. Instructors completed a post-session teaching survey to gauge fit and further refine and improve the program curriculum.

### 2.8. Statistical Analysis

All analyses were performed using STATA software, version 15.1. Means, standard deviations (SD), and frequencies were computed for all demographic data, process evaluation data, and other variables of interest. Impacts of APHL program were first evaluated by each survey item. Mean of scale scores were also calculated to measure dietary pattern (7 items), barriers of eating fruits and vegetables (5 items), perceptions regarding healthy eating (4 items), and self-efficacy in meal planning and cooking (5 items). Independent *t*-tests were conducted to compute subgroup differences in biometric outcomes for sociodemographic variables.

Methods to assess biometric outcomes: Descriptive statistics were computed as means and frequency distributions for all demographic data and outcome variables of interest. Outcome data were further categorized and evaluated (HbA1c classification [22]: <5.7%—normal, 5.7 to <6.5%—prediabetes, HbA1c ≥ 6.5%—diabetes; blood pressure classification [23]: systolic < 120 mmHg and diastolic < 80 mmHg—normal blood pressure, systolic 120–129 mmHg and diastolic < 80 mmHg—elevated blood pressure, systolic ≥ 130 mmHg or diastolic ≥ 80 mmHg—high blood pressure; BMI classification [24]: 18.5 to <25 kg/m^2^—normal, 25 to <30 kg/m^2^—overweight, ≥ 30 kg/m^2^—obese). Differences between intervention and comparison groups were tested by independent *t*-tests or chi-square tests. Outcome variables were also examined across strata of demographic subgroups. To account for repeated measures on each subject, multilevel mixed-effects regression models adjusted for ‘subject’ as a random effect were used to obtain estimations for each biometric outcome. Covariates, such as age, gender, ethnicity, and food insecurity status were added to the models to control for potential confounding effects. Group-by-time interaction terms were tested for between-group changes over time (delta). *p* ≤ 0.05 was considered statistically significant. All analyses were performed using Stata 15.1 (StataCorp, College Station, TX, USA).

Methods to assess behavioral and psychosocial outcomes: repeated measures mixed-effects linear regression models were applied to account for clustered data with time (level 1) nested in subjects (level 2). Changes in dietary behaviors, attitudes towards healthy eating, meal preparation behaviors, self-efficacy in meal planning and cooking from baseline to post-intervention were estimated. Socio-demographic variables, which included age, gender, ethnicity, education, and number of food assistance programs enrolled in by participant, were included in the models to assess for potential confounding effects. Models were selected using backward elimination methods and decisions were based on Akaike information criterion (AIC).

## 3. Results

Participant characteristics at baseline are shown in Table 3 (*n* = 33 in the APHL group, *n* = 75 in the food Rx-only group). Most participants were female (80.0%), food insecure (70.6%), and the average age was 57.0 (SD = 10.3). A majority of the participants were Hispanic, Latino American, or of Spanish origin (86.2%). Racial/ethnic composition in the APHL group was different from that in the food Rx-only group (*p* = 0.049), with fewer Hispanic in the APHL group. Otherwise, there were no statistically significant differences in baseline characteristics between the two groups. At baseline, 100% of APHL participants had HbA1c levels at or above 6.5%, while 54.5% had HbA1c levels at 9% or higher. In total, 51.7% of the APHL participants had high blood pressure, and 66.7% were classified as obese.

Table 4 demonstrates the results of the subgroup analysis for differences in biometric outcomes by sociodemographic variables. There were no differences in HbA1c by age, gender, race/ethnicity, and food insecurity status. There were significant differences in systolic BP, diastolic BP, and BMI by gender, such that females had lower BP values but conversely higher BMI as compared to males. No other differences were observed.

### 3.1. Biometric Outcome Analysis

Results of the outcome analysis showed a statistically significant pre-to-post intervention reduction in HbA1c levels among participants within the APHL group (estimated mean difference = −0.96% (−1.82, −0.10), *p*-value = 0.028). Those in the comparison group (food Rx-only) also demonstrated pre-to-post decrease in HbA1c, albeit these changes were not statistically significant (estimated mean difference = −0.48 (−1.12, 0.15), *p*-value = 0.137). Between-group changes showed a greater decrease in HbA1c among those participating in APHL as compared to food Rx-only, albeit these differences were not statistically significant (estimated mean difference = −0.48% (−1.55, 0.59), *p*-value = 0.378). No significant changes within or between-group changes were seen for systolic/diastolic blood pressure or BMI (Table 5).

### 3.2. Behavioral and Psychosocial Outcomes Analysis

A total of 41 pre-surveys (27 Spanish surveys and 14 English surveys) and 29 post-surveys (18 Spanish surveys and 11 English surveys) were analyzed.

Dietary behaviors: After the APHL program, as compared to the baseline, participants reported significant increases in servings of fruitss (Adjβ = 0.75, 95% CI: 0.34, 1.15 *p* < 0.001) and vegetables (Adjβ = 0.95, 95% CI: 0.52, 1.38, *p* < 0.001) consumed, as well as increases in the frequency of consumption of fruits (Adjβ = 0.47, 95% CI: 0.09, 0.84, *p* = 0.014) and green salad (Adjβ = 0.66, 95% CI: 0.23, 1.09, *p* = 0.003). Additionally, after the APHL program, more participants reported always eating food from each food group every day as compared to baseline (Adjβ = 0.63, 95% CI: 0.29, 0.97, *p* < 0.001).

Fruit and vegetable consumption pattern was assessed using the summative scale of 7 items (Cronbach’s alpha = 0.54) with questions asking about frequency of consumption of various foods. Pre-to-post APHL showed that there were significant increases in the scores of fruit and vegetable consumption pattern indicating improved frequency of consumption of these healthy foods among program participants (Adjβ = 0.23, 95% CI: 0.04, 0.42, *p* = 0.016).

Perceptions regarding healthy eating: When asked about perceptions of healthy eating, at post-APHL, fewer participants reported that cooking healthy food is difficult (Adjβ = −0.86, 95% CI: −1.36, −0.36, *p* = 0.001) as compared to baseline prior to APHL. None of the other variables for attitudes to healthy living changed significantly pre-to-post APHL.

Meal preparation behaviors: as compared to baseline, more participants reported significant increases in frequency of making homemade meals from scratch (Adjβ = 0.34, 95% CI: 0.08, 0.60, *p* = 0.011) and adjusting meals to be healthier (Adjβ = 0.42, 95% CI: 0.07, 0.77, *p* = 0.02) post-APHL.

Self-efficacy in meal planning and cooking: At the post-intervention survey, 82.8% of the participants rated their ability to prepare healthy meals as “excellent, very good or good”, 17.2% rated as “fair”, and none were rated as “poor”. As compared to baseline, at post-APHL, more participants reported a significant increase in confidence across most of the culinary skills, including in using knife skills in the kitchen (β = 0.56, 95% CI: 0.12, 0.99, *p* = 0.012), using basic cooking techniques (Adjβ = 0.93, 95% CI: 0.35, 1.50, *p* = 0.002), preparing root vegetables (Adjβ = 0.70, 95% CI: 0.23, 1.18, *p* = 0.004), preparing fresh or frozen green vegetables (Adjβ = 0.74, 95% CI: 0.34, 1.14, *p* < 0.001), and preparing whole grains (Adjβ = 0.96, 95% CI: 0.49, 1.44, *p* < 0.001).

Overall self-efficacy in meal planning and cooking was also assessed using a summative scale of 5 items (Cronbach’s alpha = 0.88). Pre-to-post APHL showed a significant increase in self-efficacy (confidence) in meal planning and cooking scores among program participants (Adjβ = 0.78, 95% CI: 0.39, 1.17, *p* < 0.001) (Table 6).

#### Process Evaluation

A total of *n* = 35 patients participated in the APHL program (83% participation rate), of which *n* = 27 attended 4 + sessions. Feedback was sought from patients in the comment cards after each session, allowing them to rate on usefulness of session topics and activities on a 4-point scale (1 = not useful at all, and 4 = very useful). Overall, scores across 13 session topics ranged from 2.69 to 3.03, with a mean score of 2.8 out of 4. The highest scores were for building a healthy plate and grocery shopping session topics. The range for activities, such as group discussions and goal setting were also high (ranging between 2.87 and 3.0). Interestingly, the videos scored lowest (with a range of 2.42 to 2.92 and an average of 2.56).

## 4. Discussion

Results of our pilot study demonstrated statistically significant within-group improvements in HbA1c measures among participants enrolled in APHL program (food Rx plus culinary medicine). Concurrently, there were similar changes among those in the food Rx-only group, although the magnitude of change was smaller in this group, and the changes were not statistically significant. When assessing between-group net changes, the improvements in HbA1c measures were greater among those in the APHL group than those who received food Rx-only, albeit these differences were not statistically significant, which could potentially be due to the small sample size used in our study. Future studies with a larger sample size and adequate statistical power are needed.

Food prescription programs that operate collaboratively between healthcare and social services are fast gaining popularity in the U.S., with a recent meta-analysis demonstrating significant positive impacts on obesity and diabetes outcomes [25]. However, a major limitation identified in the meta-analysis was the lack of rigor in study designs used and small sample sizes [25]. There was also significant heterogeneity in the nutrition educational components of these food prescription programs. From an intervention design perspective, the common reductionist philosophy to “eat more fruits and vegetables” ignores the complex financial, social, and environmental factors that often dictate an individual’s choice to consume a healthy dietary pattern [26,27]. Additionally, barriers such as negative taste perceptions, perceived lack of time, and the overall cost of access (purchasing, transportation, waste, etc.), are strong deterrents of consuming a healthy dietary pattern [28,29]. Thus, merely distributing healthy foods to food insecure patients in low-income communities ignores social inequalities and intrapersonal factors, such as culture, taste, self-efficacy for food preparation, and food literacy, as well as the community social support and role modeling, and thus, may not be sufficient to increase healthy food consumption [14,29]. The relatively new scientific field of culinary medicine blends the art of cooking with the science of medicine to empower patients to improve their health through the power of healthy food [12]. Our results demonstrate promise of these culinary medicine strategies used in APHL to improve the impact of food prescription programs. The pre-post changes in the intervention group in our study demonstrated significant improvements across many of the behavioral and psychosocial factors, including improved dietary patterns, perceptions towards healthy eating, improved cooking skills and meal planning and preparation behaviors, and self-efficacy towards healthy cooking and eating, that were hypothesized as mediators in our intervention mapping framework. These results concur with those of prior studies that have demonstrated significant improvements in these factors after participation in a culinary medicine curriculum. While several culinary medicine programs exist with varying adaptations, [30,31,32] to our knowledge ours is the first to be specifically designed for low-income minority patients with diabetes participating in a food prescription program.

Some of the limitations experienced in our study included the small sample size in the intervention group due to attrition, the APHL group being a convenience sample resulting in selection bias, and lack of randomization which could have resulted in confounding bias of known and unknown confounders. Furthermore, there was attrition across the intervention group for both the biometric data and the survey completion. The attrition was primarily because of communication challenges between the clinic and study staff and patients, and the inability to contact the patients in a timely manner for follow-up measures. Additionally, the behavioral and psychosocial measures were self-report, which could result in social desirability bias. These survey measures were only collected in the APHL group, without corresponding measures in the comparison group, thus limiting internal validity of these findings. Studies are currently underway using a more stringent design and a comparison group for these measures. Lastly, the pivot of the program as a result of the COVID-19 pandemic could have resulted in attenuating program impact because many of the hands-on approaches were not fully implemented virtually. The results of our study, and that of others, demonstrate the promise of culinary medicine approaches in improving biometric outcomes among high need patients, but also underscore the need for rigorous study designs and pragmatic trials to attribute changes to the intervention itself.

Strengths of our study include the successful community–clinic–academic partnerships that led to successful implementation and evaluation of this program. Given the burgeoning interest in social determinants of health, and rising rates of food insecurity, these partnerships can be scaled further. Another strength of the study was the use of validated survey items from prior studies, and leveraging on the EHR for abstracting biometric data. Finally, the pivoting of the program due to the pandemic resulted in the development of a virtual arm of the program which is a resource that can be used to reach patients who may not be able to attend the traditional in-person classes, thus adding to the existing delivery format for the program. Furthermore, despite the challenges of the pandemic and the resultant pivot, our study showed strong feasibility and acceptability of APHL program components in the participant population.

## 5. Conclusions

In conclusion, this pilot study demonstrated feasibility and promise of coupling a culinary medicine approach with food prescription programming to improve diabetes outcomes in a high need population. Plans for implementation of the APHL program using both in-person delivery and fully interactive virtual formats are currently underway. Future studies to conduct pragmatic trials using rigorous study randomized controlled trial designs and a larger sample size to test effectiveness of these strategies are warranted.

## Figures and Tables

**Table 1:** Intervention mapping framework for A Prescription for Healthy Living intervention. * Healthy food is comprised of the components of the MyPlate—which includes fruits, vegetables, legumes, healthy fats, nuts and seeds, lean proteins, whole grains, and dairy products. ** Nutrition-related chronic diseases include heart disease, stroke, high blood pressure, diabetes, and some cancers. EHR—electronic health records. SCT—social cognitive theory.

Program Inputs	Change Agents	SCT Change Objectives	Behavioral Outcomes	Physiological and Psychosocial Outcomes
APHL Training of RDN Practitioner (train-the-trainer) training (culinary skills, nutrition knowledge, program delivery) Planning and implementation support Time for RDN training APHL Materials Facilities including kitchen equipment and cooking materials Food provisions Patient curriculum Implementation manual Recipe and culinary skills toolbox APHL resources for print materials and handouts Online resources available virtually (recipe videos etc.)	Implementation Team (APHL staff, clinic dietitians, and misc. staff) Coordinates patient enrollment with EHR Plans patient curriculum implementation Provide pre and post intervention survey Patient Classes Aligns with food received from the food pantry Provide tasting of recipes prepared in class Conduct demonstration of cooking techniques Provide instruction in hands-on preparation of recipes Conduct class discussions of nutrition topics and recipe preparation Provide post-session handouts covering nutrition and cooking topics	Patients will demonstrate increase: Outcome expectations of the taste of healthy foods * Knowledge of healthy eating (appropriate type, portion size, and to manage disease condition) Self-efficacy of preparing healthy foods * Culinary skills for preparing healthy foods * Perceived social support for healthy foods * Normative beliefs of healthy foods * Healthcare Staff will demonstrate increased: Knowledge of culinary techniques Skills for the preparation of healthy foods Social support for culturally relevant foods and flavor profiles Communication with patients about preparation of healthy foods * Consistent dietary messages	Patients will increase: Healthy eating behaviors: Preparation of healthy foods at home Dietary Intake of: Vegetables Fruits Whole grains Legumes Patients will demonstrate decreased consumption of Caloric dense and nutrient deficient foods Sugar sweetened beverages Processed grains Overall caloric intake	Patients will increase: Health related quality of life Patients will decrease: Food insecurity Complications from diabetes ** HbA1c levels ** Blood pressure ** Triglyceride levels ** LDL cholesterol ** Body mass index (BMI) ** Body weight **
			Environmental Outcomes	
			Patients will demonstrate increased: Availability of healthy foods * at home Opportunity to practice healthy eating behaviors Improved home nutrition environment	

**Table 2:** APHL curriculum outline, and theoretical constructs operationalized.

A Prescription for Healthy Eating Culinary Medicine Curriculum Outline		
Five, 2 h each, hands-on sessions via Kitchen a la Cart		
Common themes for each session: Patient centered communication (e.g., facilitated discussions) Culinary skills development (e.g., knife skills, vegetable roasting) Self-efficacy building (e.g., tasting and preparing foods, picture challenges) Utilization of foods from the food pantry Group discussion and feedback		
**Session**	**Topics Covered**	**Objectives**
Session 1	MyPlate, kitchen safety, vegetable prepping (knife skills), roasting, goal setting, review patients’ recipes, and building a healthy plate activity	Participants will: (1) Describe their current barriers with healthy eating. (2) Identify a healthy plate as ½ fruits and vegetables, ¼ lean protein, and ¼ whole grains. (3) Learn to roast flavorful vegetables. (4) Learn how to use safe and effective knife skills to prepare a variety of vegetables. (5) Create a short-term goal related to building a healthy plate.
Session 2	Carbohydrate counting, label reading, whole grains, vegetable salads, goal setting, review patients’ recipes, and label reading activity	Participants will: (1) Be able to describe what foods contain carbohydrates. (2) Learn how to control glucose levels using a MyPlate approach. (3) Learn how to cook whole grain(s). (4) Practice safe and effective knife skills to prepare a variety of vegetables. (5) Refine/build from goal from session 1.
Session 3	Meal planning, grocery shopping, stir-frying & microwaving, goal setting, review patients’ recipes and meal planning, and grocery shopping activity	Participants will: (1) Discuss their success and challenges with carbohydrate counting. (2) Describe meal planning as a way to help plan the grocery list. (3) Learn how to prepare a flavorful vegetable stir-fry. (4) Learn how to microwave flavorful vegetables. (5) Refine/build from goal from session 2.
Session 4	Repurposing leftovers, meal planning, vegetable roasting, whole grains, goal setting, review patients’ recipes and planning, and repurposing activity	Participants will: (1) Discuss their challenges and successes in meal planning and grocery shopping. (2) Identify ways to plan meals in a way that repurposes leftovers. (3) Reinforce whole grain cooking and roasting vegetables. (4) Practice safe and effective knife skills to prepare a variety of vegetables. (5) Refine/build from goal from session 3.
Session 5	Eating away from home and snacking, vegetable soups and microwaving, goal setting, review patients’ recipes, and choosing healthy foods	Participants will: (1) Discuss their challenges and successes in repurposing leftovers. (2) Identify how to eat healthier meals away from the home and while snacking. (3) Learn how to prepare flavorful vegetable soups. (4) Reinforce microwave cooking and practice safe and effective knife skills. (5) Refine/build from goal from session 4.

**Table 3:** Baseline characteristics of participants, APFHL study 2020.

	Total	APFHL + Food Rx Group	Food Rx-Only Group	
	(*n* = 114)	(*n* = 35)	(*n* = 79)	
Demographics:	mean (±SD ^1^)			*p*-value ^2^
Age	55.9 (±8.9)	57.0 (±10.3)	55.4 (±8.2)	0.377
		N (%)		*p*-value ^3^
Gender				
Female	78 (68.4)	28 (80.0)	50 (63.3)	0.077
Male	36 (31.6)	7 (20.0)	29 (36.7)	
Race/ethnicity				
Hispanic, Latino American, or Spanish origin	94 (86.2)	27 (79.4)	67 (89.3)	0.049 *
Non-Hispanic	15 (13.8)	7 (20.6)	8 (10.7)	
Food insecurity status				
positive	73 (65.2)	24 (70.6)	49 (62.8)	0.428
negative	39 (34.8)	10 (29.4)	29 (37.2)	
Biometric Outcomes:	mean (±SD ^1^)			*p*-value ^2^
HbA1c	9.54 (2.15)	9.52 (2.13)	9.54 (2.17)	0.954
SBP ^4^	133.08 (19.02)	132.52 (22.80)	133.31 (17.42)	0.851
DBP ^5^	74.96 (10.64)	72.97 (12.25)	75.77 (9.88)	0.233
BMI ^6^	33.96 (7.90)	35.82 (9.45)	33.23 (7.15)	0.149
HbA1c				
Prediabetes	1 (0.09)	0 (0.0)	1 (1.4)	0.728
Diabetes				
6.5 to <9%	52 (48.2)	15 (45.5)	37 (49.3)	
≥9%	55 (50.9)	18 (54.5)	37 (49.3)	
Blood Pressure				
Normal	21 (21.0)	8 (27.6)	13 (18.3)	0.261
Elevated	15 (15.0)	6 (20.7)	9 (12.7)	
High Blood Pressure	64 (64.0)	15 (51.7)	49 (69.0)	
BMI				
Normal	5 (5.2)	1 (3.7)	4 (5.8)	0.722
Overweight	33 (34.4)	8 (29.6)	25 (36.2)	
Obese	58 (60.4)	18 (66.7)	40 (58.0)	

^1^ Standard deviation. ^2^ *p*-value was obtained from two-sample independent *t*-test ^3^ *p*-values were obtained from chi-square tests. ^4^ SBP stands for systolic blood pressure. ^5^ DBP stands for diastolic blood pressure. ^6^ BMI stands for body mass index. * significant at *p* < 0.05.

**Table 4:** Biometric outcomes at baseline by demographic groups, APHL study 2020.

	HbA1c		SBP ^1^		DBP ^2^		BMI ^3^	
	*n*	Mean (±SD)	*n*	Mean (±SD)	*n*	Mean (±SD)	*n*	Mean (±SD)
All	108	9.54 (±2.14)	100	133.08 (±19.02)	100	74.96 (±10.64)	96	33.96 (±7.90)
Age								
<49	20	10.41 (±2.15)	18	124.72 (±15.02)	18	74.39 (±9.82)	17	36.90 (±11.40)
50 to <60	52	9.59 (±2.29)	51	132.78 (±19.87)	51	74.86 (±11.04)	49	33.17 (±7.07)
≥60	36	8.98 (±1.78)	31	138.42 (±18.34)	31	75.45 (±10.73)	30	33.57(±6.60)
Gender								
Female	75	9.57 (±2.25)	67	128.93 (±17.95) *	67	72.91 (±10.54) *	65	35.22 (±8.59) *
Male	33	9.46 (±1.91)	33	141.52 (±18.58) *	33	79.12 (±9.71) *	31	31.30 (±5.42) *
Race/ethnicity								
Mexican or Chicano American	52	9.52 (±2.18)	47	132.66 (±18.73)	47	74.89 (±11.25)	45	33.62 (±7.23)
Hispanic, Latino American, or Spanish origin	38	9.88 (±2.33)	36	133.25 (±19.29)	36	73.81 (±9.77)	34	32.88 (±7.12)
Others	13	8.90 (±1.44)	13	130.85 (±21.55)	13	77.00 (±11.47)	13	36.87 (±11.55)
Food Insecurity Status								
Positive	70	9.57 (±2.29)	69	133.87 (±18.78)	69	76.07 (±10.23)	66	33.96 (±7.94)
Negative	37	9.36 (±1.78)	30	131.17 (±20.05)	30	72.20 (±11.34)	29	34.19 (±7.98)

^1^ SBP stands for systolic blood pressure. ^2^ DBP stands for diastolic blood pressure. ^3^ BMI stands for body mass index. * Statistically significant differences noted between groups, tested by independent *t*-tests.

**Table 5:** Changes in biometric outcomes, APFHL study 2020.

	Baseline		Post-Intervention		Within Group Changes ^1^		Net Changes ^1^ in Intervention Group	
	*n*	Estimated Marginal Means (95% CI ^2^)	*n*	Estimated Marginal Means (95% CI)	Marginal Differences (95% CI)	*p*-Value	Delta (95% CI)	*p*-Value
HbA1c								
APFHL + Food Rx group	33	9.60 (8.87, 10.33)	28	8.63 (7.84, 9.43)	−0.96 (−1.82, −0.10)	0.028 *	−0.48 (−1.55, 0.59)	0.378
Food Rx-only group	75	9.50 (9.01, 9.99)	50	9.02 (8.43, 9.61)	−0.48 (−1.12, 0.15)	0.137		
Systolic Blood Pressure								
APFHL+Food Rx group	29	132.57 (125.07, 140.06)	26	133.03 (125.18, 141.87)	0.46 (−7.56, 8.48)	0.217	3.90 (−5.82, 13.63)	0.431
Food Rx-only group	71	132.85 (127.99, 137.71)	55	129.40 (123.96, 134.84)	−3.44 (−8.92, 2.04)	0.798		
Diastolic Blood Pressure								
APFHL+Food Rx group	29	73.48 (69.45, 77.50)	26	70.46 (66.23, 74.68)	−3.02 (−7.55, 1.50)	0.190	−2.53 (−8.01, 2.96)	0.366
Food Rx-only group	71	75.23 (72.63, 77.84)	55	74.74 (71.80, 77.67)	−0.50 (−3.58, 2.59)	0.753		
BMI ^3^								
APFHL + Food Rx group	27	34.99 (32.20, 37.78)	22	34.77 (31.96, 37.58)	−0.22 (−1.18, 0.74)	0.649	−0.23 (−1.37, 0.91)	0.693
Food Rx-only group	69	33.55 (31.70, 35.40)	51	33.56 (31.68, 35.44)	0.007 (−0.61, 0.63)	0.982		

^1^ Adjusted estimations were obtained using multilevel mixed-effects linear model adjusted for age, gender, ethnicity, and food insecurity status. ^2^ CI stands for confidence interval. ^3^ BMI stands for body mass index. * significant at *p* < 0.05.

**Table 6:** Changes in dietary and psychosocial behavioral outcomes from baseline (*n* = 41) to post- APFHL (*n* = 29), APFHL 2020.

	Baseline	Post-APHL	Unadjusted Mixed-Effects Models ^1^	Adjusted Mixed-Effects Models ^1^
	*n* (%)	*n* (%)	β Coefficient (95% CI ^2^) *p*-Value	β coefficient (95% CI ^2^) *p*-Value
Dietary Behaviors				
Fruit consumption per day				
4 servings or more	1 (2.4)	3 (10.4)	0.75 (0.37, 1.13)	0.75 (0.34, 1.15) ^3^
2–3 servings	9 (22.0)	11 (37.9)	<0.001 *	<0.001 *
2 servings or less	31 (75.6)	15 (51.7)		
Vegetables consumption per day				
4 servings or more	1 (2.5)	5 (17.2)	1.02 (0.61, 1.43)	0.95 (0.52, 1.38) ^3^
2–3 servings	11 (26.8)	14 (48.3)	<0.001 *	<0.001 *
2 servings or less	29 (70.7)	10 (34.5)		
Whole grains consumption per day				
4 servings or more	2 (4.9)	0 (0.0)	0.52 (0.02, 1.03)	0.36 (−0.20, 0.91) ^4^
2–3 servings	7 (17.1)	8 (27.6)	0.043 *	0.210
2 servings or less	32 (78.0)	21 (72.4)		
How often do you typically eat…				
Fruit?				
Once per day or more	24 (58.5)	23 (79.3)	0.46 (0.11, 0.81)	0.47 (0.09, 0.84) ^3^
Less than once per day	17 (41.5)	6 (20.7)	0.01 *	0.014 *
Green Salad?				
Once per day or more	18 (43.9)	18 (62.1)	0.68 (0.27, 1.09)	0.66 (0.23, 1.09) ^3^
Less than once per day	23 (56.1)	11 (37.9)	0.001 *	0.003 *
French fries or other fried potatoes ^6^?				
Once per day or more	4 (10.0)	1 (3.4))	0.23 (−0.02, 0.49)	0.24 (−0.03, 0.51) ^3^
Less than once per day	36 (90.0)	28 (96.5)	0.070	0.085
Other kind of non-fried potatoes?				
Once per day or more	3 (7.5)	2 (6.9)	0.19 (−0.23, 0.60)	0.13 (−0.31, 0.57) ^3^
Less than once per day	37 (92.5)	27 (93.1)	0.385	0.568
Beans ^7^?				
Once per day or more	14 (34.2)	11 (37.9)	0.10 (−0.32, 0.52)	0.15 (−0.30, 0.59) ^3^
Less than once per day	27 (65.8)	18 (62.1)	0.636	0.525
Other non-fried vegetables ^8^?				
Once per day or more	22 (53.7)	23 (79.3)	0.55 (0.06, 1.04)	0.44(−0.05,0.92) ^3^
Less than once per day	19 (46.3)	6 (20.7)	0.027 *	0.077
How often do you eat food from each food group every day?				
Always	11 (26.8)	17 (58.6)	0.62 (0.31, 0.93)	0.63 (0.29, 0.97) ^3^
Often	16 (39.0)	10 (34.4)	<0.001 *	<0.001 *
Sometimes	9 (22.0)	1 (3.5)		
Never or rarely	5 (12.2)	1 (3.5)		
How often do you eat breakfast?				
Always	11 (27.5)	9 (32.1)	0.31 (−0.26, 0.88)	0.24 (−0.37, 0.84) ^3^
Often of sometimes	14 (35.0)	15 (53.6)	0.281	0.443
Never or rarely	15 (37.5)	4 (14.3)		
How often do you eat from a fast-food or sit-down restaurant ^9^?				
More than once per week	7 (17.5)	6 (20.7)	0.02 (−0.36, 0.39)	0.03 (−0.37, 0.43)^3^
Once per week or less	20 (50.0)	10 (34.5)	0.923	0.883
Not at all	13 (32.5)	13 (44.8)		
Subscale for dietary pattern:	mean (±SD)	mean (±SD)		
Dietary Pattern ^10^	3.44 (±0.50)	3.72 (±0.49)	0.26 (0.07, 0.44)	0.23 (0.04, 0.42) ^3^
(Cronbach’s alpha: 0.54)			0.006 *	0.016 *
Perceptions regarding healthy food				
Healthy food tastes bad or bland.				
Agree	10 (24.4)	4 (13.8)	−0.38 (−0.97, 0.20)	−0.42(−1.05, 0.21) ^3^
Neutral	8 (19.5)	4 (13.8)	0.200	0.191
Disagree	23 (56.1)	21 (72.4)		
Cooking healthy food takes too much time.				
Agree	6 (15.0)	3 (10.3)	−0.21 (−0.73, 0.32)	−0.17 (−0.73, 0.39) ^3^
Neutral	9 (22.5)	5 (17.3)	0.443	0.555
Disagree	25 (62.5)	21 (72.4)		
Buying healthy food is too expensive for me.				
Agree	25 (61.0)	15 (51.7)	−0.28 (−0.85, 0.29)	−0.29 (−0.90, 0.33) ^3^
Neutral	5 (12.2)	5 (17.3)	0.338	0.357
Disagree	11 (26.8)	9 (31.0)		
Cooking healthy food is difficult.				
Agree	7 (17.5)	0 (0.0)	−0.84 (−1.32, −0.37)	−0.86 (−1.36, −0.36) ^3^
Neutral	8 (20.0)	1 (3.4)	<0.001 *	0.001 *
Disagree	25 (62.5)	28 (96.6)		
Subscale:	mean (±SD)	mean (±SD)		
Perceptions regarding healthy eating ^11^ (Cronbach’s alpha: 0.74)	2.52 (±1.17)	2.08 (±0.85)	−0.44 (−0.87, −0.01)	−0.45 (−0.91, 0.009) ^3^
			0.043 *	0.055
Perceived Barriers of eating fruits and vegetables				
I don’t eat fruits and vegetables as much as I like to because…				
...they cost too much.				
Agree	14 (34.2)	12 (41.4)	0.14 (−0.38, 0.67)	0.25 (−0.31, 0.81) ^4^
Neutral	11 (26.8)	5 (17.2)	0.593	0.382
Disagree	16 (39.0)	12 (41.4)		
…they are easily spoiled.				
Agree	14 (35.0)	6 (20.7)	−0.60 (−1.14, −0.05)	−0.52 (−1.05, 0.02) ^3^
Neutral	10 (25.0)	4 (13.8)	0.032 *	0.057
Disagree	16 (40.0)	19 (65.5)		
...they take too much time to prepare.				
Agree	7 (17.5)	4 (13.8)	−0.32 (−0.87,0.24)	−0.30 (−0.89, 0.29) ^3^
Neutral	8 (20.0)	3 (10.3)	0.263	0.326
Disagree	25 (62.5)	22 (75.9)		
…the restaurants I go to don’t serve them.				
Agree	5 (12.5)	3 (10.4)	−0.28 (−0.77, 0.20)	−0.28 (−0.81, 0.24) ^3^
Neutral	14 (35.0)	7 (24.1)	0.256	0.290
Disagree	21 (52.5)	19 (65.5)		
…I don’t know how to cook the vegetables.				
Agree	5 (12.5)	3 (10.3)	−0.18 (−0.63, 0.28)	−0.19 (−0.68, 0.30) ^3^
Neutral	5 (12.5)	4 (13.8)	0.443	0.450
Disagree	30 (75.0)	22 (75.9)		
Subscale:	mean (±SD)	mean (±SD)		
Barriers of eating fruits and vegetables ^12^ (Cronbach’s alpha: 0.61)	2.45 (±0.95)	2.15 (±0.80)	−0.26(−0.51,−0.008)	−0.24 (−0.50, 0.02) ^3^
			0.043 *	0.076
Meal Preparation Behaviors				
Fruit and vegetable portion when thinking about preparing a plate of meal.				
One half	24 (58.5)	20 (69.0)	0.09 (−0.12, 0.31)	0.02 (−0.19, 0.24) ^3^
One fourth or three fourth	17 (41.5)	9 (31.0)	0.395	0.820
How often do you engage in the following behaviors?				
Compare price before buying food.				
Always	14 (34.2)	17 (58.6)	0.51 (0.08, 0.95)	0.36 (−0.07, 0.78) ^3^
Often	10 (24.3)	5 (17.2)	0.021 *	0.100
Sometimes	14 (34.2)	5 (17.2)		
Never or rarely	3(7.3)	2 (6.9)		
Plan meals ahead of time.				
Always	15 (36.6)	15 (51.7)	0.34 (−0.10, 0.79)	0.29 (−0.17, 0.75) ^3^
Often	10 (24.4)	5 (17.2)	0.131	0.214
Sometimes	10 (24.4)	7 (24.1)		
Never or rarely	6 (14.6)	2 (6.9)		
Use grocery list when you go shopping.				
Always	15 (36.6)	16 (55.2)	0.56 (0.0005, 1.11)	0.41 (−0.11, 0.93) ^5^
Often	11 (26.8)	7 (24.1)	0.050	0.119
Sometimes	7 (17.1)	5 (17.2)		
Never or rarely	8 (19.5)	1(3.5)		
Worry that your food might run out before you get money to buy more.				
Always	8 (20.0)	9 (32.1)	−0.04 (−0.60, 0.51)	0.04 (−0.54, 0.61) ^4^
Often	8 (20.0)	4 (14.3)	0.884	0.904
Sometimes	17 (42.5)	7 (25.0)		
Never or rarely	7 (17.5)	8 (28.6)		
Use the “nutrition facts” on food labels.				
Always	14 (34.1)	11 (37.9)	0.32 (−0.19, 0.84)	0.46 (−0.006, 0.92) ^3^
Often	2 (4.9)	5 (17.2)	0.215	0.053
Sometimes	12 (29.3)	7 (24.2)		
Never or rarely	13 (31.7)	6 (20.7)		
Make homemade meals “from scratch”.				
Always	20 (50.0)	20 (69.0)	0.36 (0.11, −0.61)	0.34 (0.08, 0.60) ^3^
Often	10 (25.0)	6 (20.8)	0.005 *	0.011 *
Sometimes	7 (17.5)	1 (3.4)		
Never or rarely	3 (7.5)	2(6.9)		
Adjust meals to be more healthy.				
Always	15 (36.6)	17 (58.6)	0.42 (0.10, 0.75)	0.42 (0.07, 0.77) ^3^
Often	15 (36.6)	8 (27.6)	0.01 *	0.02 *
Sometimes	7 (17.1)	3 (10.3)		
Never or rarely	4 (9.8)	1 (3.5)		
Self-efficacy in meal planning and cooking				
How sure were you that you could:				
Use knife skills in the kitchen.				
Extremely Sure/very sure	21 (72.4)	28 (96.6)	0.76 (0.27, 1.25)	0.56 (0.12, 0.99) ^3^
Neutral	3 (10.4)	1 (3.4)	0.003 *	0.012 *
Not very sure/not at all sure	5 (17.2)	0 (0.0)		
Use basic cooking techniques.				
Sure	16 (55.2)	27 (93.1)	1.03 (0.45, 1.62)	0.93 (0.35, 1.50) ^5^
Neutral	5 (17.2)	2 (6.9)	0.001 *	0.002 *
Not sure	8 (27.6)	0(0.0)		
Prepare root vegetables.				
Extremely Sure/very sure	20 (69.0)	25 (86.2)	0.76 (0.29, 1.23)	0.70 (0.23, 1.18) ^4^
Neutral	2 (6.9)	2 (6.9)	0.002 *	0.004 *
Not very sure/not at all sure	7 (24.1)	2 (6.9)		
Prepare fresh or frozen green vegetables.				
Extremely Sure/very sure	23 (79.3)	28 (96.6)	0.83 (0.43, 1.23)	0.74 (0.34, 1.14) ^3^
Neutral	2 (6.9)	1 (3.4)	<0.001 *	<0.001 *
Not very sure/not at all sure	4 (13.8)	0 (0.0)		
Prepare whole grains.				
Extremely Sure/very sure	19 (65.5)	28 (96.6)	1.14 (0.64, 1.64)	0.96 (0.49, 1.44) ^3^
Neutral	2 (6.9)	1 (3.4)	<0.001 *	<0.001 *
Not very sure/not at all sure	8 (27.6)	0 (0.0)		
Subscale:	mean (±SD)	mean (±SD)		
Self-efficacy in meal planning and cooking ^13^ (Cronbach’s alpha: 0.88)	3.69 (±0.97)	4.59 (±0.59)	0.90 (0.49, 1.32)	0.78 (0.39, 1.17) ^3^
			<0.001 *	<0.001 *

^1^ Dependent variables with original 5-point likert scales were used in each model. ^2^ Confidence interval. ^3^ Adjusted for ethnicity and education. ^4^ Adjusted for gender, ethnicity, and education. ^5^ Adjusted for ethnicity, education, and number of food assistance programs. ^6^ Other fried potatoes such as home fries, hash browns, or tater tots. ^7^ Beans such as refried beans, baked beans, pinto beans, black beans, or other cooked beans, but do not count green beans or string beans. ^8^ Other non-fried vegetables such as carrots, broccoli, green beans. ^9^ Consider breakfast, lunch, and dinner. ^10^ Sum scores of 7 likert scale variables, regarding how often do you eat fruits, green salad, french fries, non-fried potatoes, beans, non-fried vegetables, and eating out. Possible scores: 1–5, 5 = more than once a day, 1 = not at all. ^11^ Sum scores of 4 likert scale variables regarding perceptions on healthy eating. Possible scores: 1–5, 5 = strongly agree, 1 = strongly disagree. ^12^ Sum scores of 5 likert scale variables regarding perceived barriers of eating fruits and vegetables. Possible scores: 1–5, 5 = strongly agree, 1 = strongly disagree. ^13^ Sum scores of 5 likert scale variables regarding self-efficacy in meal preparation and cooking. Possible scores: 1–5, 5 = strongly agree, 1 = strongly disagree. * Findings statistically significant at *p* ≤ 0.05.

## Data Availability

Restrictions apply to the availability of these data. Patient consent was not obtained for sharing of data with third party, and hence are not available.

## References

[1] U.S. Department of Health and Human Services, Centers for Disease Control and Prevention (2020). National Diabetes Statistics Report 2020 Estimates of Diabetes and its Burden in the United States. https://www.cdc.gov/diabetes/pdfs/data/statistics/national-diabetes-statistics-report.pdf.

[2] Rowley W.R., Bezold C., Arikan Y., Byrne E., Krohe S. (2017). Diabetes 2030: Insights from yesterday, today, and future trends. Popul. Health Manag..

[3] Hu E.A., Steffen L.M., Coresh J., Appel L.J., Rebholz C.M. (2020). Adherence to the healthy eating index-2015 and other dietary patterns may reduce risk of cardiovascular disease, cardiovascular mortality, and all-cause mortality. J. Nutr..

[4] Zhang F., Liu J., Rehm C. (2018). Trends and Disparities in Diet Quality among US Adults by Supplemental Nutrition Assistance Program Participation Status. https://jamanetwork.com/journals/jamanetworkopen/fullarticle/2684625.

[5] Dean O., Hartline-Grafton H. (2017). The Impact of Poverty, Food Insecurity, and Poor Nutrition on Health and Well-Being.

[6] Raghupathi W., Raghupathi V. (2018). An empirical study of chronic diseases in the united states: A visual analytics approach to public health. Int. J. Environ. Res. Public Health.

[7] Marko D., Linder S.H., Reynolds T.F., Tullar J.M. (2019). Self-reported Health Status in Houston Area, Health of Houston Survey 2018 Fact Sheet.

[8] Houston State of Health: Adults Who Are Overweight or Obese. Updated 2020. http://www.houstonstateofhealth.com/indicators/index/view?indicatorId=56&localeId=2675.

[9] Hager E.R., Quigg A.M., Black M.M., Coleman S.M., Heeren T., Rose-Jacobs R., Cook J.T., de Cuba S.A.E., Casey P.H., Chilton M. (2010). Development and validity of a 2-item screen to identify families at risk for food insecurity. Pediatrics.

[10] McWhorter J.W., Raber M., Sharma S.V., Moore L.S., Hoelscher D.M. (2019). The nourish program: An innovative model for cooking, gardening, and clinical care skill enhancement for dietetics students. J. Acad. Nutr. Diet..

[11] Eldredge L.K.B., Markham C., Ruiter R., Fernandez M., Kok G., Parcel G. (2016). Planning Health Promotion Programs: An Intervention Mapping Approach.

[12] La Puma J. (2016). What is culinary medicine and what does it do?. Popul. Health Manag..

[13] Polak R., Phillips E.M., Nordgren J., La Puma J., La Barba J., Cucuzzella M., Graham R., Harlan T., Burg T., Eisenberg D. (2016). Health-related culinary education: A summary of representative emerging programs for health professionals and patients. Glob. Adv. Health Med..

[14] Kuehn B.M. (2019). Heritage diets and culturally appropriate dietary advice may help combat chronic diseases. JAMA.

[15] Sicker K., Habash D., Hamilton L., Nelson N.G., Robertson-Boyd L., Shaikhkhalil A.K. (2020). Implementing culinary medicine training: Collaboratively learning the way forward. J. Nutr. Educ. Behav..

[16] Evert A.B., Dennison M., Gardner C.D., Garvey W.T., Lau K.H.K., MacLeod J., Mitri J., Pereira R.F., Rawlings K., Robinson S. (2019). Nutrition therapy for adults with diabetes or prediabetes: A consensus report. Diabetes Care.

[17] Bandura A. (1986). Social Foundations of Thought and Action: A Social Cognitive Theory.

[18] Yaroch A.L., Tooze J., Thompson F.E., Blanck H.M., Thompson O.M., Colón-Ramos U., Shaikh A.R., McNutt S., Nebeling L.C. (2012). Evaluation of three short dietary instruments to assess fruit and vegetable intake: The national cancer institute’s food attitudes and behaviors survey. J. Acad. Nutr. Diet..

[19] Parks C.A., Uvena L., Quam J., Garvin T.M., Yaroch A. (2015). Development and testing of a revised cooking matters for adults survey. Am. J. Health Behav..

[20] Condrasky M.D., Williams J.E., Catalano P.M., Griffin S.F. (2011). Development of psychosocial scales for evaluating the impact of a culinary nutrition education program on cooking and healthful eating. J. Nutr. Educ. Behav..

[21] Bowling A. (2005). Just one question: If one question works, why ask several?. J. Epidemiol. Community Health.

[22] American Diabetes Association Understanding A1C. https://www.diabetes.org/a1c.

[23] American Heart Association Editorial Staff Understanding Blood Pressure Readings. https://www.heart.org/en/health-topics/high-blood-pressure/understanding-blood-pressure-readings.

[24] Centers for Disease Control and Prevention About Adult BMI. Updated 2020. https://www.cdc.gov/healthyweight/assessing/bmi/adult_bmi/index.html.

[25] Bhat S., Coyle D.H., Trieu K., Neal B., Mozaffarian D., Marklund M., Wu J.H. (2021). Healthy food prescription programs and their impact on dietary behavior and cardiometabolic risk factors: A systematic review and meta-analysis. Adv. Nutr..

[26] Darmon N., Drewnowski A. (2015). Contribution of food prices and diet cost to socioeconomic disparities in diet quality and health: A systematic review and analysis. Nutr. Rev..

[27] Mozaffarian D. (2017). Foods, obesity, and diabetes-are all calories created equal?. Nutr. Rev..

[28] Neumark-Sztainer D., Wall M., Perry C., Story M. (2003). Correlates of fruit and vegetable intake among adolescents. findings from project EAT. Prev. Med..

[29] Story M., Kaphingst K.M., Robinson-O’Brien R., Glanz K. (2008). Creating healthy food and eating environments: Policy and environmental approaches. Annu. Rev. Public Health.

[30] Olfert M.D., Wattick R.A., Hagedorn R.L. (2020). Experiential application of a culinary medicine cultural immersion program for health professionals. J. Med. Educ. Curric. Dev..

[31] Hie S.A. Analysis of a Pilot Study of a Culinary Medicine Course for Community Health Workers—ProQuest. Updated 2020. https://www.proquest.com/docview/2470670012?pq-origsite=gscholar&fromopenview=true.

[32] Barkoukis H., Swain J., Rogers C., Harris S.R. (2019). Culinary medicine and the RDN: Time for a leadership role. J. Acad. Nutr. Diet..

